# Transcriptome analysis of *Nicotiana benthamiana* infected by *Tobacco curly shoot virus*

**DOI:** 10.1186/s12985-018-1044-1

**Published:** 2018-09-03

**Authors:** Ke Li, Gentu Wu, Mingjun Li, Mingge Ma, Jiang Du, Miao Sun, Xianchao Sun, Ling Qing

**Affiliations:** College of Plant Protection, Southwest University, Chongqing, 400716 People’s Republic of China

**Keywords:** Begomovirus, *Tobacco curly shoot virus*, Betasatellite, *Nicotiana benthamiana*, RNA-seq

## Abstract

**Background:**

*Tobacco curly shoot virus* (TbCSV) is a monopartite begomovirus associated with betasatellite (Tobacco curly shoot betasatellite*,* TbCSB), which causes serious leaf curl disease on tomato and tobacco in China. It is interesting that TbCSV induced severe upward leaf curling in *Nicotiana benthamiana*, but in the presence of TbCSB, symptoms changed to be downward leaf curling. However, the mechanism of interactions between viral pathogenicity, host defense, viral-betasatellite interactions and virus-host interactions remains unclear.

**Methods:**

In this study, RNA-seq was used to analyze differentially expressed genes (DEGs) in *N. benthamiana* plants infected by TbCSV (Y35A) and TbCSV together with TbCSB (Y35AB) respectively.

**Results:**

Through mapping to *N. benthamiana* reference genome, 59,814 unigenes were identified*.* Transcriptome analysis revealed that a total of 4081 and 3196 DEGs were identified in Y35AB vs CK (control check) and Y35A vs CK, respectively. Both GO and KEGG analyses were conducted to classify the DEGs. Ten of the top 15 GO terms were enriched in both DEGs of Y35AB vs CK and Y35A vs CK, and these enriched GO terms mainly classified into three categories including biological process, cellular component and molecular function. KEGG pathway analysis indicated that 118 and 111 pathways were identified in Y35AB vs CK and Y35A vs CK, respectively, of which nine and six pathways were significantly enriched. Three major pathways in Y35AB vs CK involved in metabolic pathways, carbon metabolism and photosynthesis, while those in Y35A vs CK were related to Ribosome, Glyoxylate and dicarboxylate metabolism and DNA replication. We observed that 8 PR genes were significantly up-regulated and 44 LRR-RLK genes were significantly differentially expressed in Y35A treatment or in Y35AB treatment. In addition, 7 and 13 genes were identified to be significantly changed in biosynthesis and signal transduction pathway of brassinosteroid (BR) and jasmonic acid (JA) respectively.

**Conclusions:**

These results presented here would be particularly useful to further elucidate the response of the host plant against virus infection.

**Electronic supplementary material:**

The online version of this article (10.1186/s12985-018-1044-1) contains supplementary material, which is available to authorized users.

## Background

*Tobacco curly shoot virus* (TbCSV), a monopartite begomovirus (genus *Begomovirus*, family *Geminiviridae*), was isolated from tobacco in Yunnan province, China in 2002 [1]. Some TbCSV isolates were identified associating with a betasatellite molecule (Tobacco curly shoot betasatellite*,* TbCSB) [2]. TbCSV is one of the most important pathogens causing leaf curl disease on tomato and tobacco, and is a severe constraint to crop yields. TbCSV consists of a circular single-stranded DNA genome that encodes 6 proteins, including AV1 (coat protein, CP) and AV2 in virion-sense strand whilst AC1 (replication-related, Rep), AC2 (transcriptional activator, TrAP), AC3 (replication enhancer, REn) and AC4 in complementary-sense strand [1]. TbCSB is a small circular single-stranded DNA molecule which encodes a sole protein βC1 [2].

Viral infection is a complicated procedure involving in the interaction between viruses and host plants. Understanding host responses to viral infection is advantageous in the development of effective strategies for virus control. In recent years, the interactions between viruses and host plants were clarified through analysis of transcriptomics [3–5]. RNA-seq is a recently developed approach to transcriptome profiling via deep-sequencing technologies, which provides a far more precise measurement of levels of transcripts and their isoforms than other methods [6]. Thus, this technique was widely applied in interpretation the interaction between virus infection and host plant. The multiple resistance mechanisms against cotton leaf curl disease (CLCuD) were revealed by the transcriptome analysis based on an RNA-seq in a naturally immune cotton species (*Gossypium arboreum*) caused by CLCuD [7]. The technology of transcriptomics and proteomics were employed to study the differential host gene expression during *Mungbean yellow mosaic India virus* (MYMIV) and *Mungbean yellow mosaic virus* (MYMV) infection under natural conditions [8]. The differential regulations of host genes revealed to be involved in cell cycle, cell-wall biogenesis, chloroplast, photosynthesis, hormone and sulphur assimilation pathways which may contribute to symptom development in soybean plants [8]. Recently, a research performed RNA-seq-based transcriptome sequencing of *Jatropha curcas mosaic virus*-infected and healthy leaf tissues of *J. curcas*, which provides a repertoire of molecular components after viral infection [9]. Similarly, several host genes were identified to be involved in different cellular and physiological processes during the infection of other viruses, including *South African cassava mosaic virus* (SACMV) [10], *Tomato yellow leaf curl Sardinia virus* (TYLCSV), *Mungbean yellow mosaic India virus* (MYMIV) [11, 12], and *Chilli leaf curl virus* (ChiLCV) [13].

At present, it has been reported that the infectious clone of TbCSV induced severe upward leaf curling in *N. benthamiana*, but in the presence of TbCSB the symptom changed to a downward leaf curl [14]. Thus, TbCSV may represent an evolutionary intermediate between the truly monopartite begomoviruses and those that require the association of betasatellite [14]. Moreover, studies showed that AC2 and AC4 proteins of TbCSV mediate suppression of RNA silencing [15], and βC1 protein of TbCSB could bind single- and double- stranded DNA to suppress host RNA silencing activities [16]. However, it remains unclear that the molecular mechanism of viral pathogenicity, host defense and viral-host interactions. In this study, in order to get insights into the molecular mechanisms in response to TbCSV infection in *N. benthamiana* plants, the transcriptome analysis of *N. benthamiana* infected by TbCSV or TbCSV/TbCSB was performed, including differentially expressed genes, GO enrichment, KEGG enrichment, pathogenesis-related (PR) protein genes, LRR-RLK genes, brassinosteroid and jasmonic acid pathways. These data contribute to a better knowledge on the molecular mechanisms of TbCSV- and TbCSV/TbCSB- host interaction, and serve as a basis for devising new strategies to control TbCSV/TbCSB disease complex.

## Methods

### Virus sources and agroinoculation

The infectious clones of TbCSV isolate Y35 (pBinY35A-1.9) (Y35A) and its betasatellite (pBinY35β-2.0) (Y35B) were provided by Professor Xueping Zhou in the Biotechnology Institute of Zhejiang University. Both viral infectious clones were introduced into *Agrobacterium tumefaciens* strain EHA105. *N. benthamiana* (accession: Nb-1) plants were grown in a greenhouse under a 16 h light and 8 h dark cycle at 26 °C. The *Agrobacterium* with infectious clones was infiltrated into *N. benthamiana* leaves at 4–6 leaf stage as previously described [2].

### DNA extraction and PCR detection

Leaf samples were collected at 20 days-post inoculation (dpi) and total DNA were extracted using CTAB method [17]. The TbCSV-specific primers (TbCSV-F, 5′-ATGCCTCAGCCAAGAAAACTTTT-3′; TbCSV-R, 5′-TCAACACGACGACGTCTGTTCCC-3′) and TbCSB-specific primers (TbCSB-F, 5′-ATGACAATTAAATACAACAACAAG-3′; TbCSB-R, 5′-TCATACATTAGCTATTGTCCC-3′) were designed to amplify fragments of 1086 bp and 357 bp in size, respectively. PCR reactions were performed in a 20 μL volume with reaction mixtures containing 10 μL of 2 × Taq Master Mix (Novoprotein Scientific Inc., Shanghai, China), 0.5 μL of DNA, 0.25 μM of each primer and a proper volume of ddH_2_O. The PCR reaction conditions were as follows: 94 °C for 3 min, followed by 35 cycles of denaturation for 30 s at 94 °C, annealing for 30 s at 52 °C, extension for 30–65 s at 72 °C (depending on primer pairs used in distinct reactions), and a final extension for 10 min at 72 °C.

### Materials for RNA sequencing

Leaf tissues of *N. benthamiana* plants were sampled at 20 dpi, and were immediately frozen and ground in liquid nitrogen and stored at − 80 °C. After PCR detection, RNA-seq was performed for stored samples. Three treatments were setting as follows: plants infected with TbCSV alone (Y35A treatment); plants infected with TbCSV and TbCSB (Y35AB treatment); uninfected plants as control (CK). There are three biological replicates per treatment respectively.

### RNA extraction and cDNA synthesis

Total RNA was extracted from *N. benthamiana* leaf tissues with RNAiso Plus (TAKARA Bio, Inc) as manufacturer’s protocol. All RNA samples were first treated with gDNA Eraser (TAKARA Bio, Inc) and reverse-transcribed using a Prime Script RT reagent Kit (Perfect Real Time) according to manufacturer’s instructions.

### Library preparation for transcriptome sequencing

A total amount of 3 μg RNA per sample was used as input for RNA sample preparations. Sequencing libraries were generated using NEBNext® Ultra™ RNA Library Prep Kit for Illumina® (NEB, USA) following manufacturer’s recommendations and index codes were added to attribute sequences to each sample. Briefly, mRNA was purifed by using poly-T oligo-attached magnetic beads. After fragmentation, the cDNA was synthesized, and NEBNext Adaptors with hairpin loop structures were ligated to prepare for hybridization. PCR products were purifed (AMPure XP system), and library quality was assessed by Agilent Bioanalyzer 2100 system [18]. Finally, nine libraries were successfully constructed, and were sequenced using HiSeq™ 2500 equipment (Illumina, SanDiego, CA, USA) by 50 bp single-end (SE50) methods.

### Quality control

For sequence quality control, raw data (raw reads) of fastq format were firstly processed through in-house perl scripts. In this step, clean data (clean reads) were obtained by removing reads containing adapter, reads containing ploy-N and low quality reads from raw data. Meanwhile, Q20, Q30, GC-content and sequence duplication level of clean data were calculated. All the downstream analyses were based on the clean data with high quality [19].

### Reads mapping to the reference genome

The draft sequence of the *N. benthamiana* reference genome have been downloaded from the SGN ftp site (ftp://ftp.solgenomics.net/genomes/Nicotiana_benthamiana/assemblies) directly [20]. Index of the reference genome was built using Bowtie v2.2.3 (Broad Institute, Cambridge, MA, USA) [21] and single-end clean reads were aligned to the reference genome using TopHat v2.0.12 (Broad Institute, Cambridge, MA, USA) (mismatch = 2) [22]. The TopHat was selected as the mapping tool for that TopHat can generate a database of splice junctions based on the gene model annotation file and thus a better mapping result than other non-splice mapping tools [23].

### Quantification of gene expression level

HTSeq v0.6.1 was used to count reads numbers mapped to each gene [24]. And then FPKM (Fragments per kilobase of transcript sequence per millions base pairs sequenced) of each gene was calculated based on the length of the gene and reads count mapped to this gene. FPKM, expected number of Fragments Per Kilobase of transcript sequence per Millions base pairs sequenced, considers the effect of sequencing depth and gene length for the reads count at the same time, and is currently the most commonly used method for estimating gene expression levels [25].

### Differential gene expression analysis

Differential expression analysis of Y35AB vs CK and Y35A vs CK was performed using the DESeq R package (1.18.0) (parameters: negative binomial distribution-based statistic, BH-FDR corrected *p*-value < 0.05) [26], which provides statistical routines for determining differential expression in digital gene expression data using a model based on the negative binomial distribution. The resulting *p*-values were adjusted using the Benjamini and Hochberg’s approach for controlling the false discovery rate (FDR).

### GO term and KEGG pathway enrichment analysis

Gene Ontology (GO; http://www.geneontology.org) enrichment analysis of differentially expressed genes (DEGs) was implemented by the GOseq R package (corrected *p*-value < 0.05) [27], in which gene length bias was corrected. GO terms with corrected *p*-value less than 0.05 were considered significantly enriched by differential expressed genes. REVIGO was used for analysis of the enriched GO terms (http://revigo.irb.hr). KEGG (http://www.genome.jp/kegg) is a database resource for understanding high-level functions and utilities of the biological system from molecular-level information, especially large-scale molecular datasets generated by genome sequencing and other high-throughput experimental technologies. And KOBAS software was used to test the statistical enrichment of differential expression genes in KEGG pathways (corrected *p*-value < 0.05) [28].

### Quantitative real-time PCR validation

To validate the RNA-seq data, quantitative real-time PCR (RT-qPCR) was conducted to examine the selected pattern of DEGs. For reverse transcription, 1 μg of total RNA was treated with gDNA Eraser (TAKARA Bio, Inc) and reverse-transcribed using a Prime Script RT reagent Kit (Perfect Real Time) and oligo (dT) as the primer according to the manufacturer’s protocol. Primer sets were designed using AlleleID software (v6.0) (Additional file 1: Table S1). RT-qPCR was conducted by using NovoStart® SYBR qPCR Super Mix Plus (Novoprotein, Shanghai, China) on CFX 96 Real-Time System (Bio-Rad). Actin was used as a reference for calculating relative abundances using the 2^-△△Ct^ method [29]. All RT-qPCR experiments were performed in triplicate.

## Results

### Plants with typical viral symptoms in Y35A and Y35AB treatments

**Fig. 1:**
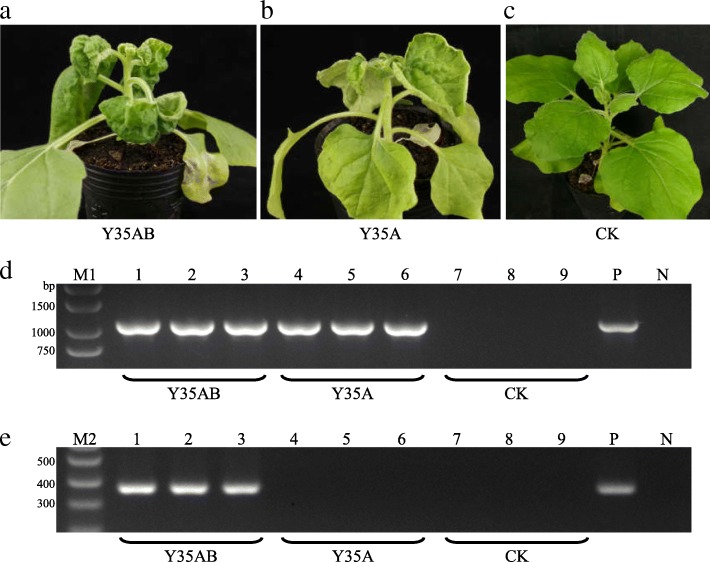
Comparison of *N. benthamiana* plant symptoms of virus-infected with healthy control at 20 dpi. **a** Co-inoculation of TbCSV and TbCSB (Y35AB). **b** Solitary inoculation of TbCSV (Y35A). **c** Healthy control (CK). **d** Specific detection of TbCSV DNA-A in the nine plants for sequencing by PCR with primers TbCSV-F/TbCSV-R. **e** Specific detection of TbCSB in the nine plants for sequencing by PCR with primers TbCSB-F/TbCSB-R. M1: BM2000 + 1.5 kb DNA Marker; M2: DNA Marker I; 1–3: Y35AB plants; 4–6: Y35A plants; 7–9: CK plants; P: positive control; N: negative control, respectivly

At 20 dpi, Y35A inoculated plants showed slight upward leaf curling and Y35AB inoculated plants showed severe downward leaf curl (Fig. 1a-c). PCR detection showed that they are consistent with expectations (Fig. 1d-e). Symptom observation and virus detection results showed that these plants were reliable for follow-up RNA-seq. Leaf tissues at same positions were collected from every treatment for RNA-seq.

### Overview of transcriptome sequencing

To profile gene expression during virus infection, RNA-seq libraries were constructed for the control- and virus-inoculated *N. benthamiana* plants. 5.76 GB clean bases data and 59,814 unigenes were obtained from nine samples. There were 11834496–13118731, 12537134–15503750 and 11290015–12633203 clean reads in the three treatments of Y35AB, Y35A, and CK. The proportion of total reads that mapped to *N. benthamiana* genome of each sample was more than 96.09%, and that of uniquely mapped was above 72.69%. More than 95.58% of the clean reads had quality scores at the Q30 (an error probability for base calling of 0.1%) level [30]. Furthermore, the GC content ranges from 43.58 to 44.38%. The sequencing data are summarized in Table 1.

**Table 1:** Summary of the results of RNA-seq data

Sample name	Y35AB-1	Y35AB-2	Y35AB-3	Y35A-1	Y35A-2	Y35A-3	CK-1	CK-2	CK-3	mean
Raw reads	12166453	11965470	13272880	15527372	12560210	14214740	12646000	11302705	11794017	12827761
Clean reads	12032720	11834496	13118731	15503750	12537134	14192135	12633203	11290015	11782562	12769416
Clean bases	0.60G	0.59G	0.66G	0.78G	0.63G	0.71G	0.63G	0.56G	0.59G	0.64
Total mapped	11620770	11438690	12605297	14934455	12075773	13732342	12346163	11139553	11524248	12379699
	96.58%	96.66%	96.09%	96.33%	96.32%	96.76%	97.73%	98.67%	97.81%	96.99%
Uniquely mapped	8961324	8847537	9609766	11389720	9176512	10320807	9238644	8316046	8564617	9380553
	74.47%	74.76%	73.25%	73.46%	73.19%	72.72%	73.13%	73.66%	72.69%	73.48%
Q30 (%)	95.74	95.68	95.58	96.20	96.08	96.14	95.76	95.70	95.80	95.85
GC contents (%)	43.58	43.75	44.11	44.15	44.23	44.38	43.93	43.80	43.92	43.98

Note: Clean reads: Reads from sequencing after filtering low-quality reads. Clean bases: The number of clean reads is multiplied by the length and converted to G. Q30: The percentage of bases with a Phred value >30

### Analysis of DEGs

**Fig. 2:**
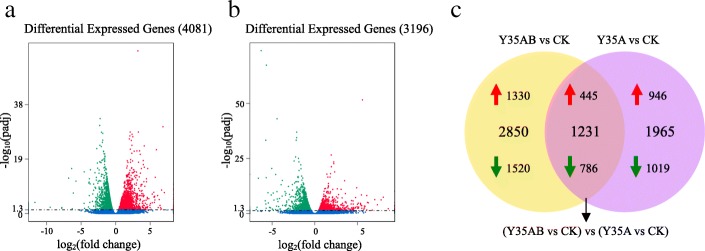
Volcano plots and Venn diagrams of DEGs (**a**-**b**). DEGs of Y35AB vs CK and Y35A vs CK displayed by volcano plots. The abscissa shows the fold change difference in the expression of genes in different groups, and the vertical coordinates indicate the adjusted *p*-values for the differences in expression. Genes without significant differences are indicated by blue dots. The up-regulated genes are represented by red dots, and the down-regulated genes are represented by green dots. Venn diagrams showing the overlap in DEGs between Y35AB vs CK and Y35A vs CK (**c**)

An adjusted *p*-value < 0.05 by DESeq was used to identify DEGs. In the Y35AB vs CK, 4081 DEGs (Fig. 2a, Additional file 2: Table S2), including 1775 up-regulated and 2306 down-regulated genes, were identified (Fig. 2c). And 3196 transcripts (1391 up-regulated and 1805 down-regulated genes) showed significant changes in Y35A vs CK (Fig. 2b, c and Additional file 3: Table S3). However, a total of 1232 DEGs in Y35AB vs CK overlap with those in Y35A vs CK, including 445 up-regulated and 786 down-regulated genes (Fig. 2c).

### GO enrichment analysis

**Fig. 3:**
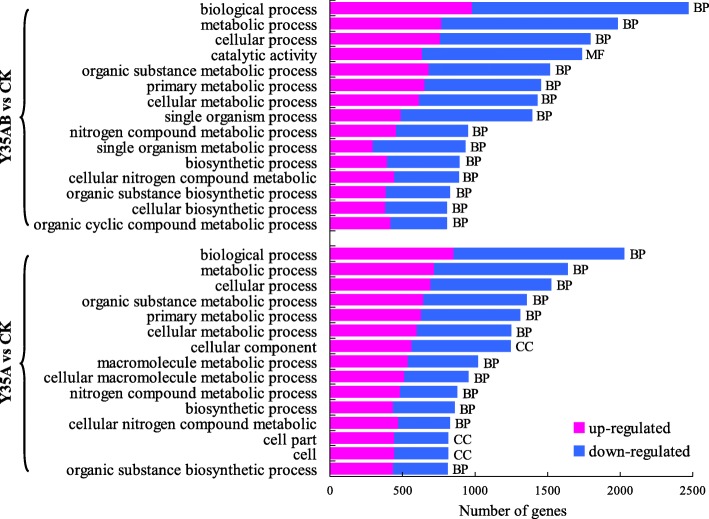
Most of the top 15 GO terms of three categories significantly enriched in DGEs of Y35AB vs CK and Y35A vs CK. MF, molecular function; BP, biological process; CC, cellular component

Gene Ontology, an internationally standardized gene function classification system, was used to classify the DEGs. The results were enriched in Y35AB vs CK and Y35A vs CK, and 124 and 76 of them were significantly enriched, respectively. Further, the top 15 GO functional annotation terms were listed, and 10 of them were shared between Y35AB vs CK and Y35A vs CK, associated with biological process, metabolic process, cellular process, organic substance metabolic process, primary metabolic process, cellular metabolic process, nitrogen compound metabolic process, biosynthetic process, cellular nitrogen compound metabolic process and organic substance biosynthetic process (Fig. 3, Additional files 4 and 5: Tables S4 and S5). Furthermore, it was found that these enriched GO terms mainly classified into biological process.

### KEGG pathway enrichment analysis

**Fig. 4:**
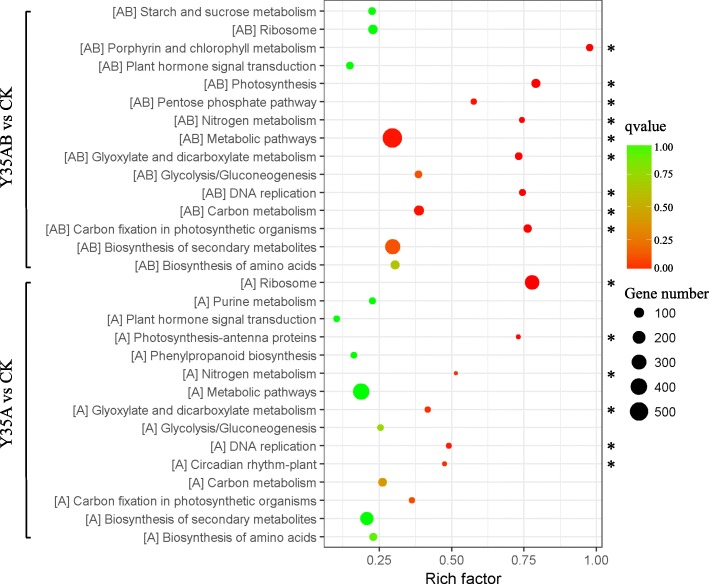
KEGG pathways of the more or main significantly enriched in DGEs. The rich factor reflects the degree of enriched DGEs in a given pathway. The number of enriched DGEs in the pathway is indicated by the circle area, and the circle color represents the ranges of the corrected *p*-value

In order to further clarify molecular and biological functions of the genes, these DEGs were mapped to the KEGG database. KEGG pathway analysis indicated that 118 and 111 pathways were identified in Y35AB vs CK and Y35A vs CK, respectively, of which nine and six were significantly enriched with *p-*value< 0.05. The significantly enrichment analysis showed that DEGs of Y35AB vs CK involved in metabolic pathways, carbon metabolism, photosynthesis, carbon fixation in photosynthetic organisms, glyoxylate and dicarboxylate metabolism, porphyrin and chlorophyll metabolism, DNA replication, pentose phosphate pathway and nitrogen metabolism. While, DEGs involved in ribosome, glyoxylate and dicarboxylate metabolism, DNA replication, circadian rhythm-plant, photosynthesis-antenna proteins and nitrogen metabolism were significantly enriched in Y35A vs CK (Fig. 4, Additional files 6 and 7: Tables S6 and S7).

Metabolic pathway was the major pathway which contains the largest number of DEGs in both Y35AB vs CK and Y35A vs CK, but DEGs involved in metabolic pathway were not significantly different in Y35A vs CK, it further indicated that the presence of TbCSB differentially changed metabolic pathways in *N. benthamiana.* The rich factor of porphyrin and chlorophyll metabolism pathway in Y35AB vs CK was near 1.00, the results showed that almost all genes in this pathway differentially expressed in TbCSV associated with betasatellite infected plants. Nevertheless, ribosome pathway might play an important role in response to TbCSV infection for the number of enriched DEGs in ribosome pathway wasnear to that enriched in metabolic pathways, and the rich factor was more than 0.75.

### Screening of Y35AB vs CK and Y35A vs CK common highly expressed DEGs

FPKM reflecting both the effect of sequencing depth and gene length for the read count, and is a commonly used method for estimating gene expression levels [31]. The fold change in gene expression was used to distinguish differentially expressed genes between samples because of the significance of digital gene expression profiles [32]. The intersection of highly expressed DGEs in Y35AB vs CK and Y35A vs CK was considered. Finally, 17 DEGs were identified (|log_2_(Fold change)| ≥ 1 and FPKM≥50) from significantly enriched KEGG pathways, these 17 genes were shared between Y35AB vs CK and Y35A vs CK all down-regulated, and involved in six pathways, including photosynthesis, porphyrin and chlorophyll metabolism, metabolic pathways, carbon metabolism, carbon fixation in photosynthetic organisms, and glyoxylate and dicarboxylate metabolism. Additionally, six DEGs that not enriched in any pathway were screened (|log_2_ (Fold change) | ≥ 2 and FPKM≥50), including four up-regulated and two down-regulated. In total, 23 candidate genes were screened as shown in Table 2.

**Table 2:** Screened candidate genes in Y35AB and Y35A

Gene symbol	Unigene ID	Protein properties	log_2_FC^a^	log_2_FC^b^	pathway
*PsaFa*	Niben101Scf 00271g04024	photosystem I reaction center subunitIII, chloroplastic-like	-1.4180	-1.1769	Photosynthesis
*PsaFb*	Niben101Scf 02156g05001	photosystem I reaction center subunit III, chloroplastic-like	-1.5190	-1.6335	Photosynthesis
*PsaFc*	Niben101Scf 04964g00002	photosystem I reaction center subunit III, chloroplastic-like	-1.5257	-1.2865	Photosynthesis
*PsaNa*	Niben101Scf 17701g01020	photosystem I reaction center subunit N, chloroplastic-like	-1.6702	-1.5537	Photosynthesis
*PsaNb*	Niben101Scf 35628g00001	photosystem I reaction center subunit N, chloroplastic-like	-1.3129	-1.0396	Photosynthesis
*ChlHa*	Niben101Scf 04388g00011	magnesium-chelatase subunit ChlH, chloroplastic-like	-1.6623	-2.1794	Porphyrin and chlorophyll metabolism
*ChlHb*	Niben101Scf 07214g00015	magnesium-chelatase subunit ChlH, chloroplastic-like	-1.4638	-1.8585	Porphyrin and chlorophyll metabolism
*BchP*	Niben101Scf 06249g03002	geranylgeranyl diphosphate reductase, chloroplastic-like	-1.8576	-1.0521	Porphyrin and chlorophyll metabolism
*HemA*	Niben101Scf 10063g00003	glutamyl-tRNA reductase 1,chloroplastic-like	-1.4101	-2.0903	Porphyrin and chlorophyll metabolism
*DXS*	Niben101Scf 00246g04005	probable 1-deoxy-D-xylulose-5-phosphate synthase, chloroplastic-like	-1.0040	-1.0126	Metabolic pathways
*GME1-X1*	Niben101Scf 01696g06050	GDP-mannose 3,5-epimerase 1-like,transcript variant X1	-1.6022	-1.1137	Metabolic pathways
*CAB3A*	Niben101Scf 03961g00004	chlorophyll a-b binding protein 50, chloroplastic-like	-1.1418	-1.0719	Metabolic pathways
*GDCSa*	Niben101Scf 02480g02012	glycine dehydrogenase (decarboxylating), mitochondrial-like	-1.5732	-1.1698	Carbon metabolism
*GDCSb*	Niben101Scf 03839g04019	glycine dehydrogenase (decarboxylating), mitochondrial-like	-1.1991	-1.0395	Carbon metabolism
*rbcS*	Niben101Scf 02381g04022	ribulose bisphosphate carboxylase small chain S41, chloroplastic-like	-1.2691	0.7603	Carbon fixation in photosynthetic organisms
*FBA1*	Niben101Scf 02864g04008	fructose-bisphosphate aldolase 1,chloroplastic-like	-1.3167	-1.0072	Carbon fixation in photosynthetic organisms
*glsF*	Niben101Scf 04198g01002	ferredoxin-dependent glutamate synthase, chloroplastic-like	-1.1161	-1.0704	Glyoxylate and dicarboxylate metabolism
*GRP*	Niben101Scf 03052g00011	glycine-rich protein-like	2.1379	1.4647	No pathway enriched
*GRP3a*	Niben101Scf 01084g05013	glycine-rich protein 3-like	3.9423	3.1735	No pathway enriched
*GRP3b*	Niben101Scf 01084g03004	glycine-rich protein 3-like	2.0529	1.8836	No pathway enriched
*CAX3*	Niben101Scf 01329g00002	vacuolar cation/proton exchanger 3-like	-2.2799	-1.2535	No pathway enriched
*FSD1*	Niben101Scf 03679g03009	Fe superoxide dismutas ,chloroplastic-like	-2.1527	-1.9924	No pathway enriched
*PU*	Niben101Scf 10524g02015	putative uncharacterized protein	2.0616	0.9243	No pathway enriched

*FC* Fold change

^a^Y35AB vs. CK

^b^Y35A vs. CK

### Validation of the selected genes by RT-qPCR

**Fig. 5:**
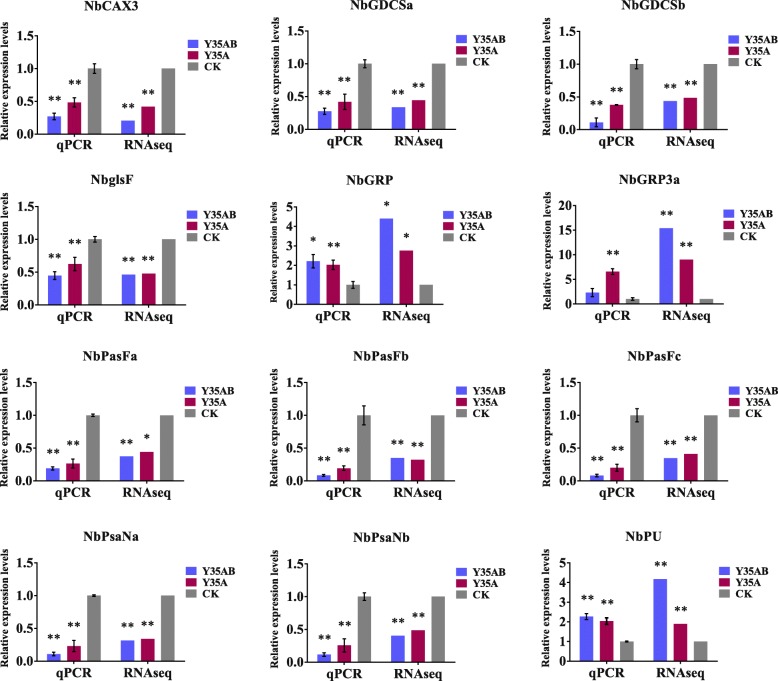
Comparison of relative expression levels determined by RNA-seq and qPCR on 12 genes selected. All qPCR reactions were used three biological replica samples, repeated three times, and the data are presented as the mean ± SD. Relative expression levels are calculated from Ct values according to the 2^–ΔΔCt^ method. Actin was the reference gene for these qPCR experiments. Asterisks indicate statistically significant differences compared with CK, “*” indicate significant difference (0.01 ≤ *p* < 0.05), “**” indicate extremely significant difference (*P* < 0.01)

To validate the RNA-seq data, 12 genes were selected randomly for RT-qPCR analysis from the Table 2, including genes *GDCSa*, *GDCSb*, *PsaFa*, *PsaFb*, *PsaFc*, *PsaNa*, *PsaNb*, *glsF*, *CAX3*, *GRP*, *GRP3a* and *PU*. The results indicated that the genes expression patterns by RT-qPCR were consistent with that by transcriptome sequencing (Fig. 5), the fold changes between RNA-seq and RT-qPCR were different which maybe caused by the sensitive of methodology or the used samples between RNA-seq and RT-qPCR were not the same one.

### Analysis of pathogenesis-related (PR) genes

An important feature of the plant defense in response to pathogen attack is the induction and accumulation of various PR proteins which are also a part of systematic acquired resistance (SAR). There were 29 PR genes identified by a local BLAST in RNA-seq dataset and online BLAST analysis based on reported PR family genes in *N. tabacum*. Among them, eight genes (27.6%) were significantly up-regulated, including *PR-2* (Glucan endo-1,3-beta-glucosidase-like), *PR-3* (Chitinase 8, glycoside hydrolase), *PR-5* (Pathogenesis-related thaumatin superfamily protein), *PR-11* (Chitinase-3-like protein 2), *PR-17* (Plant basic secretory protein family protein), of them four genes were found in Y35AB vs CK, and five genes were observed in Y35A vs CK (Table 3).

**Table 3:** Screened PRP family genes

PR family	Unigene ID	Protein properties	log_2_FC^a^	qvalue^a^	log_2_FC^b^	qvalue^b^
PR1	Niben101Scf13926g01014	Cysteine-rich secretory protein, allergen V5/Tpx-1-related				
	Niben101Scf03376g03004	Cysteine-rich secretory protein, allergen V5/Tpx-1-related				
	Niben101Scf00107g03008	Cysteine-rich secretory protein, allergen V5/Tpx-1-related				
	Niben101Scf01999g07002	Cysteine-rich secretory protein, allergen V5/Tpx-1-related				
PR2	Niben101Scf01001g00005	Glucan endo-1,3-beta-glucosidase-like, Glycoside hydrolase, family 17				
	Niben101Scf01001g00004	Glucan endo-1,3-beta-glucosidase-like, Glycoside hydrolase, family 17				
	Niben101Scf01001g00003	Glucan endo-1,3-beta-glucosidase-like, Glycoside hydrolase, family 17			Inf	0.023496
	Niben101Ctg13736g00004	Glucan endo-1,3-beta-glucosidase-like, Glycoside hydrolase, family 17			Inf	1.94E-04
	Niben101Scf04869g03002	Glucan endo-1,3-beta-glucosidase-like, Glycoside hydrolase, family 17	3.9490	0.038646	5.4381	2.14E-04
	Niben101Scf01001g00006	Glucan endo-1,3-beta-glucosidase-like, Glycoside hydrolase, family 17				
PR3	Niben101Scf02041g00002	Chitinase 8, Glycoside hydrolase, family 19			5.1058	1.99E-05
PR4	Niben101Scf01400g00014	Thaumatin-like protein				
	Niben101Scf03436g01016	Thaumatin-like protein				
PR5	Niben101Scf00126g00008	Pathogenesis-related thaumatin superfamily protein	0.9949	0.027102		
	Niben101Scf05554g05006	Pathogenesis-related thaumatin superfamily protein	1.4585	3.24E-08		
PR6	Niben101Scf00953g05001	Cysteine-rich secretory protein, allergen V5/Tpx-1-related				
	Niben101Scf04053g01004	Cysteine-rich secretory protein, allergen V5/Tpx-1-related				
PR9	Niben101Scf03460g04004	Peroxidase 53, Haem peroxidase				
	Niben101Scf07182g05012	Peroxidase 53, Haem peroxidase				
PR10	Niben101Scf03526g00006	Major pollen allergen Bet v 1-M/N, Bet v I type allergen				
	Niben101Scf10735g00016	Major pollen allergen Bet v 1-M/N, Bet v I type allergen				
	Niben101Scf02474g01024	Major pollen allergen Bet v 1-M/N, Bet v I type allergen				
	Niben101Scf01938g04007	Major pollen allergen Bet v 1-M/N, Bet v I type allergen				
PR11	Niben101Scf06295g04023	Chitinase-3-like protein 2, Glycoside hydrolase superfamily	4.4843	0.007133		
	Niben101Scf01789g04010	Chitinase-3-like protein 2, Glycoside hydrolase superfamily				
PR17	Niben101Scf03385g02011	Plant basic secretory protein family protein, uncharacterised protein family			2.7696	6.69E-04
	Niben101Scf03385g01006	Plant basic secretory protein family protein, uncharacterised protein family				
	Niben101Scf01341g01002	Plant basic secretory protein family protein, uncharacterised protein family				
	Niben101Ctg10643g00004	Plant basic secretory protein family protein, uncharacterised protein family				

log_2_FC > 0, up-regulated, log_2_FC < 0, down-regulated. Inf: the readcount of control (CK) was zero. Blank lattices show genes expression has no significant difference

*FC* Fold change

^a^Y35AB vs. CK

^b^Y35A vs. CK

### Analysis of LRR-RLKs genes

Leucine-rich repeat receptor-like protein kinases (LRR-RLKs) are the largest of receptor-like kinases in plants and play crucial roles in development and stress responses. In the present study, We combined RNA-seq dataset obtained LRR-RLK sequences with previously NCBI reported sequences to perform analysis, results showed that a total of 71 unigenes were identified potential LRR-RLK genes in RNA-seq dataset, however, 44 significantly different unigenes were observed. Among them, 18 unigenes were found in Y35AB vs CK with 10 unigenes down-regulated and the other 8 unigenes up-regulated, and 32 unigenes expressed in Y35A vs CK, with 20 and 12 unigenes were down-regulated and up-regulated, respectively. Five unigenes were commonly regulated in both Y35AB vs CK and Y35A vs CK, including one up-regulated and four down-regulated unigenes (Table 4).

**Table 4:** Screened DEGs of LRR-RLKs

Unigene ID	Protein properties	log_2_FC^a^	qvalue^a^	log_2_FC^b^	qvalue^b^
Niben101Scf13404g00002	Brassinosteroid LRR receptor kinase-like; BRI1			-0.8361	0.000272
Niben101Scf07123g01015	Elicitor-inducible leucine-rich repeats receptor-like protein			5.5080	0.000289
Niben101Scf00104g02013	F-box/LRR-repeat protein 17-like			0.8664	0.021066
Niben101Scf04252g00009	F-box/LRR-repeat protein 17-like			1.8551	0.003213
Niben101Scf09559g00005	F-box/LRR-repeat MAX2 homolog A-like	0.8581	0.018415		
Niben101Scf15394g00006	F-box/LRR-repeat protein 14-like	-0.8927	0.013552		
Niben101Scf20268g00001	LRR receptor-like serine/threonine-protein kinase ERECTA			-0.7116	0.011214
Niben101Scf05301g03002	LRR receptor-like serine/threonine-protein kinase ERL1	-0.7516	0.013687	-0.7589	0.013627
Niben101Scf00247g01007	LRR receptor-like serine/threonine-protein kinase ERL1			-0.5633	0.024694
Niben101Scf11723g02003	LRR receptor-like serine/threonine-protein kinase ERL1	-0.6679	0.026512	-0.7100	0.017084
Niben101Scf03619g03004	LRR receptor-like serine/threonine-protein kinase FEI2	-0.5426	0.018415		
Niben101Scf03925g01010	LRR receptor-like serine/threonine-protein kinase GSO1	2.3343	0.007479	3.1551	0.041202
Niben101Scf02203g04006	Plant intracellular Ras-group-related LRR protein 4-like			-0.6640	0.048845
Niben101Scf00206g00014	putative F-box/LRR-repeat protein At5g02700	1.5307	0.020135		
Niben101Scf00073g04006	probable LRR receptor-like serine/threonine-protein kinase At1g07650			-1.4546	0.005161
Niben101Scf00244g03004	probable LRR receptor-like serine/threonine-protein kinase At1g06840	-0.6603	0.021347		
Niben101Scf00418g02007	probable LRR receptor-like serine/threonine-protein kinase At4g37250			-0.5934	0.047527
Niben101Scf00920g09027	probable LRR receptor-like serine/threonine-protein kinase At5g48740	-1.1084	0.000766		
Niben101Scf01061g08014	probable LRR receptor-like serine/threonine-protein kinase At4g20940			-1.0192	0.001468
Niben101Scf01278g09008	probable LRR receptor-like serine/threonine-protein kinase At1g56140			0.9239	0.023952
Niben101Scf02357g08010	probable LRR receptor-like serine/threonine-protein kinase At1g07650	-0.6424	0.001803		
Niben101Scf02665g15003	probable LRR receptor-like serine/threonine-protein kinase At1g06840	-0.5769	0.044996		
Niben101Scf02827g07005	probable LRR receptor-like serine/threonine-protein kinase At3g47570			2.3756	0.027087
Niben101Scf03098g00011	probable LRR receptor-like serine/threonine-protein kinase At1g34110			-1.2224	3.35E-06
Niben101Scf03262g02006	probable LRR receptor-like serine/threonine-protein kinase At2g24230			-0.6865	0.030882
Niben101Scf03445g05005	probable LRR receptor-like serine/threonine-protein kinase At4g26540			-0.8802	0.000669
Niben101Scf03735g07013	probable LRR receptor-like serine/threonine-protein kinase At4g36180	2.0409	0.002356		
Niben101Scf05348g01026	probable LRR receptor-like serine/threonine-protein kinase At1g12460			-1.3373	3.02E-05
Niben101Scf05405g07003	probable LRR receptor-like serine/threonine-protein kinase At3g47570			2.0599	0.003076
Niben101Scf05405g07006	probable LRR receptor-like serine/threonine-protein kinase At3g47570			1.5589	0.022376
Niben101Scf05767g05006	probable LRR receptor-like serine/threonine-protein kinase At4g20940			-0.8478	0.000497
Niben101Scf05977g01008	probable LRR receptor-like serine/threonine-protein kinase At1g12460			-1.2826	8.11E-05
Niben101Scf06144g00013	probable LRR receptor-like serine/threonine-protein kinase At4g20940			-0.9557	7.81E-05
Niben101Scf06151g02014	probable LRR receptor-like serine/threonine-protein kinase At1g67720	0.7683	0.046840		
Niben101Scf06562g02013	probable LRR receptor-like serine/threonine-protein kinase At4g26540	-0.5968	0.021640	-0.9943	2.01E-05
Niben101Scf06789g03005	probable LRR receptor-like serine/threonine-protein kinase At1g67720	0.9483	0.042093	0.9711	0.042650
Niben101Scf07034g06018	probable LRR receptor-like serine/threonine-protein kinase At4g36180			-0.8501	0.034812
Niben101Scf07515g02006	probable LRR receptor-like serine/threonine-protein kinase At1g56140			0.6846	0.005558
Niben101Scf07681g01013	probable LRR receptor-like serine/threonine-protein kinase At4g20940			-0.7450	0.014076
Niben101Scf07695g01023	probable LRR receptor-like serine/threonine-protein kinase At4g30520	0.7628	0.001146		
Niben101Scf07736g01007	probable LRR receptor-like serine/threonine-protein kinase At1g63430			0.6791	0.006612
Niben101Scf08134g02008	probable LRR receptor-like serine/threonine-protein kinase At4g37250	-0.5193	0.047055	-0.7817	0.000797
Niben101Scf10101g00023	probable LRR receptor-like serine/threonine-protein kinase At4g30520	0.6651	0.002463		
Niben101Scf11047g00001	probable LRR receptor-like serine/threonine-protein kinase At2g16250			0.7640	0.047280

log_2_FC > 0, up-regulated, log_2_FC < 0, down-regulated. Blank lattices show genes expression has no significant difference

*FC* Fold change

^a^Y35AB vs. CK

^b^Y35A vs. CK

### Analysis of brassinosteroid and jasmonic acid pathway

**Fig. 6:**
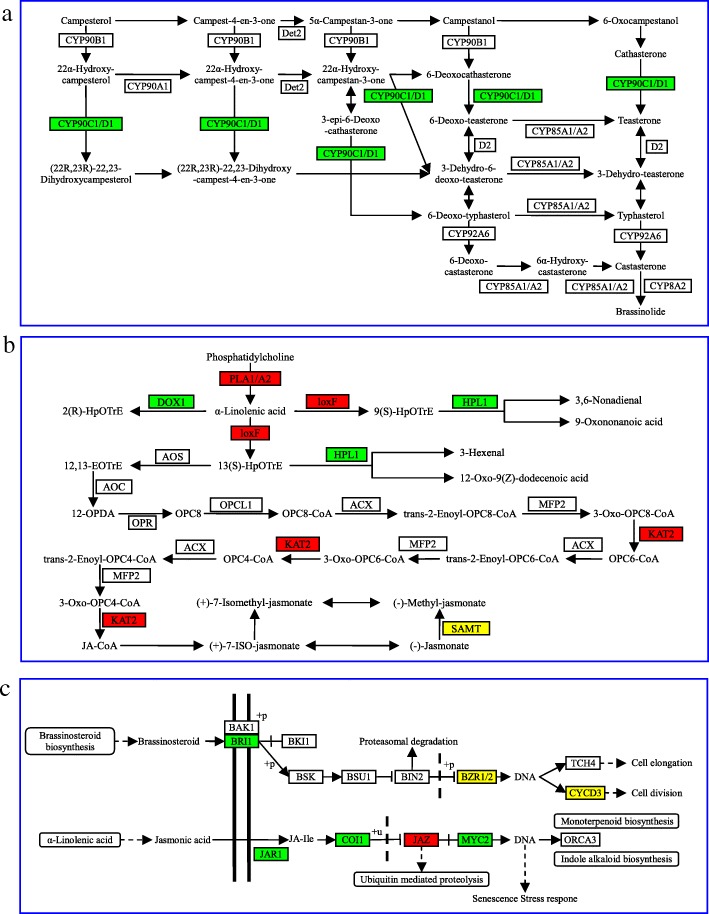
DEGs of BR and JA in biosynthesis and signal transduction pathway. **a** BR biosynthesis pathway, **b** JA biosynthesis pathway, **c** signal transduction pathway of BR and JA, respectively. Genes with red background were up-regulated, green background were down-regulated, yellow background some were up-regulated and some were down-regulated

Plant hormones are not only essential for growth and development, but also play crucial roles in plant defense responses [33, 34]. The transcriptome data showed that only one brassinosteroid (BR) synthesis-related gene, 3-epi-6-deoxocathasterone 23-monooxygenase (*CYP90C1/D1*) was significantly decreased upon virus infection (Fig. 6a), it is likely result in a reduction of brassinosteroid. By contrast, expressions of jasmonic acid (JA) -ralated biosynthesis genes *DOX1*, *HPL1*, *SAMT-X1* were decreased and *PLA1/A2*, *loxF*, *KAT2*, *SAMT-X2* were significantly increased upon virus infection (Fig. 6b). We conjecture that the expression level of jasmonic acid might was increase. In addition, part of BR and JA signaling genes expression level were also altered, *BRI1*, *BZR2*, *CYCD3-X2* were down-regulated and *BZR1*, *CYCD3-X1* were up-regulated in BR signal transduction pathway, and *JAR1*, *COI1*, *MYC2* were down- regulated and *JAZ1*, *JAZ2* were up-regulated in JA signal transduction pathway (Fig. 6c). All synthesis- and signal transduction- related significantly difference expressed unigenes of BR and JA pathway shown in Table 5.

**Table 5:** Significant difference expression unigenes of BR and JA pathway

	Gene symbol	Unigene ID	Protein properties	log_2_FC^a^	qvalue^a^	log_2_FC^b^	qvalue^b^
BR	*CYP90C1/D1*	Niben101Scf05764g04013	3-epi-6-deoxocathasterone 23-monooxygenase	-0.8193	0.019172		
	*BRI1*	Niben101Scf13404g00002	Brassinosteroid LRR receptor kinase-like, BRI1			-0.8361	2.72E-04
	*BZR1*	Niben101Scf03110g05009	Brassinosteroid resistant 1	0.5592	0.025611		
	*BZR2*	Niben101Scf06112g01006	Brassinosteroid resistant 2			-1.2497	7.36E-07
	*CYCD3-X1*	Niben101Scf05643g01012	Cyclin D3, 3 protein, plant	1.3753	1.44E-07		
	*CYCD3-X1*	Niben101Scf02445g10017	Cyclin D3, 3 protein, plant	1.2863	0.002511		
	*CYCD3-X2*	Niben101Scf00107g02002	Cyclin D3, 2 protein, plant	-0.8767	0.017675	-1.2925	2.43E-04
JA	*PLA1/A2*	Niben101Scf00711g02004	Triacylglycerol lipase SDP1-like; Phospholipase A1/A2	1.8227	0.003036		
	*DOX1*	Niben101Scf07070g02002	Alpha-dioxygenase	-3.8126	7.60E-13	-3.9498	7.95E-14
	*loxF*	Niben101Scf05407g00001	Linoleate 9S-lipoxygenase-like	0.9407	0.021540		
	*HPL1*	Niben101Scf00313g08016	Fatty acid hydroperoxide lyase, HPL1	-1.2170	1.14E-09	-0.9629	7.37E-07
	*HPL1*	Niben101Scf02207g11009	Fatty acid hydroperoxide lyase, HPL1	-1.4966	1.06E-05		
	*KAT2*	Niben101Scf10189g02009	3-ketoacyl-CoA thiolase 2, peroxisomal			0.6201	0.007053
	*SAMT-X1*	Niben101Scf05122g00005	Salicylate carboxymethyltransferase-like	-1.4545	2.97E-11	-0.8505	2.96E-04
	*SAMT-X2*	Niben101Scf01146g12006	Salicylate carboxymethyltransferase-like			1.6487	0.010833
	*JAR1*	Niben101Scf01076g00005	Jasmonic acid-amino synthetase	-0.6477	0.003456	-0.8340	0.001705
	*COI1*	Niben101Scf02280g08005	Coronatine-insensitive 1, COI1	-0.6123	0.024317	-0.8990	2.81E-04
	*JAZ1*	Niben101Scf00298g01006	Jasmonate ZIM-domain protein 1	0.7577	0.039355		
	*JAZ2*	Niben101Scf07798g03026	Jasmonate ZIM domain-containing protein			0.7728	3.95E-04
	MYC2	Niben101Scf15224g00002	Transcription factor MYC2			-0.6238	0.042856

log_2_FC > 0, up-regulated, log_2_FC < 0, down-regulated. Blank lattices show genes expression has no significant difference

*FC* Fold change

^a^Y35AB vs. CK

^b^Y35A vs. CK

## Discussion

TbCSV is a serious threat to many crops in China. In this study, RNA-seq -based transcriptomic characterization and comparative analysis of TbCSV-infected *N. benthamiana* with control plants was used to shed some light on understanding the molecular interactions of this pathosystem. 4081 and 3196 DEGs were screened from Y35AB vs CK and Y35A vs CK, respectively. Among the top 15 enriched physiological pathways, the porphyrin and chlorophyll metabolism, photosynthesis and pentose phosphate pathway were significantly affected in Y35AB vs CK. It suggested that, the disorder of these pathways might be responsible to the more serious leaf curling symptom on Y35AB-infected *N. benthamiana* plants. In addition, previous reports showed that βC1, an essential pathogenicity protein encoded by betasatellite, is required for symptom induction and RNA silencing suppression [2, 16, 35–37]. Thus, βC1 protein might contribute to the specific changes of those genes or biological pathways in Y35AB-infected plants.

In our study, the DEGs expression with high levels that mainly concerned with photosynthesis-related genes including photosynthesis, porphyrin and chlorophyll metabolismand carbon fixation in photosynthetic organisms were down-regulated in *N. benthamiana* plants infected with TbCSV. Meanwhile, the KEGG pathway analysis showed significant enrichments of the photosynthesis, and the porphyrin and chlorophyll metabolism pathway in Y35AB-infected plants, with higher rich factor. It has been reported that photosynthesis-related genes could be affected upon virus infection such as *Tobacco mosaic virus* (TMV) [38], *Cucumber mosaic virus* (CMV) [39], *Rice stripe virus* (RSV) [40, 41] and *Alfalfa mosaic virus* (AMV) [42]. And the chloroplast, one of the most dynamic organelles ofplant cell, plays an active part in the defense response and is crucial for inter organelle signaling [43]. Therefore, viruses need to suppress the chloroplast-mediated defense by employing pathogenicity factors, such as effector proteins, for successful infections [44, 45]. Furthermore, a previous study showed that a Radish leaf curl betasatellite (RaLCB) damages the structural and functional integrity of chloroplasts, leading to inhibition of photosynthesis and symptom formation [46]. Some photosynthesis-related genes were down-regulated during both Y35A and Y35AB infections, indicating that these genes might participate in the interaction between TbCSV and *N. benthamiana*.

An important feature of the plant defense response to pathogen attack is the induction and accumulation of various PR proteins, which are also a part of systematic acquired resistance (SAR) [47]. Previously, PR proteins were divided into five families based on their localization, isoelectric point, molecular mass, and biological activity [48]. PR proteins are currently categorized into 17 families. At present, besides *PR-6* is predicted by automated computational analysis based on a genomic sequence (gene ID: LOC107768382), nine out of 17 families, including *PR-1*, *PR-2* [49], *PR-3*, *PR-4*, *PR-5* [50], *PR-9* [51], *PR-10* [52], *PR-11* [53], *PR-17* [54] families were reported that associated with the acquired resistance to pathogen infections. Among these PR proteins, *PR-1* is generally considered as a marker gene of disease resistance [55]. Based on NCBI retrieval and local BLAST analysis, 29 *PR* genes in *N. benthamiana* were identified. In the present study, the expression levels of 8 *PR* genes were found to be significantly regulated in virus-infected *N. benthamiana* plants (Table 3). It was previously reported that in pathogens-infected wheat, the genes encoding peroxidase, *PR-1*, *PR-2*, *PR-3*, *PR-4*, and *PR-5* were induced after 6–12 h and reached the highest levels at 36–48 h [56, 57]. In our research, leaf tissues of *N. benthamiana* plants for RNA-seq assays were collected at 20 dpi, and the transcript levels of some PR genes (8 out of 29) were regulated significantly. For subsequent research, the expression of *PR* genes can be determined in a time course study. Based on our research, we speculated that some types of PR proteins might play roles in defense response to TbCSV infection.

In plants, many cellular signaling transductions are mediated by receptor-like kinases (RLKs). Leucine-rich repeat receptor-like protein kinases (LRR-RLKs) are the largest group of receptor-like kinases in plants and play crucial roles in development and stress responses [58]. LRR-RLKs regulate a wide range of plant growth and development, including meristem development [59, 60], secondary growth [61], microsporogenesis and embryogenesis [62]. Besides, some LRR-RLKs mediated plant resistance against bacterial [63, 64] or viral pathogens [65, 66]. In this study, 71 LRR-RLKs genes were identified. Among them, 44 were differentially expressed in virus-infected *N. benthamiana* plants. The results showed that these LRR-RLKs genes might play important roles in *N. benthamiana* responses to TbCSV infection. By contrast, in the present of TbCSB, less number of *LRR-RLK*s genes were regulated (Table 4).

BR and JA are two important plant hormones that play crucial roles in plant growth and development [67] and also involved in plant defense responses to pathogen infections [68–70]. In the BR signaling pathway, BRASSINAZOLE RESISTANT (BZR) is a transcription factor, which orchestrating plant developmental and physiological processes by regulating BR-target gene expression [71, 72]. BRASSINOSTEROID INSENSITIVE 1 (BRI1) is a brassinosteroid receptor, of which ubiquitously expresses plasma membrane-localized protein kinase [73], belonging to an LRR-RLKs subfamily [74]. For the JA signaling transduction, the perception of JA is mediated by the co-receptor CORONATINE INSENSITIVE 1 (COI1), which is an F-box protein cooperated with a family of the JASMONATE ZIM-domain (JAZ) transcriptional proteins. Upon the pathogens infection, bioactive JAs promote direct interaction between COI1 and JAZ proteins and then trigger the SCF-COI1 complex, resulting in poly-ubiquitination and degradation of the JAZ proteins. Ultimately, a variety of transcription factors could be activated for the expression of the JA-responsive genes [75]. Besides, MYC2 is a bHLH (basic helix-loop-helix) transcription factor and a positive regulator of JA responses [76]. A study has shown that rhizobacterium-mediated induction of JA reduces the symptoms of CMV infection in *Arabidopsis thaliana* ecotype Columbia plants [77]. In this study, *BRI1*, *BZR1*, *BZR2*, *CYCD3*, *JAR1*, *COI1*, *JAZ* and *MYC2* genes were significantly regulated under the infection of TbCSV (Fig. 6). It suggested that BR and JA pathways played a role in response to TbCSV infection.

## Conclusion

In this study, a genome-wide transcript profile was obtianed for *N. benthamiana* plant infected by TbCSV or plus TbCSB using RNA-seq approaches, and genes involved in plant defense system were found to be significantly regulated after TbCSV and TbCSV /TbCSB infection. The information provided in our study would be particularly useful for investigating the molecular mechanisms concerning begomovirus-host interaction and excavate resistance genes.

## Additional files

Additional file 1: **Table S1.** Primers used for RT-qPCR. (DOCX 18.9 kb)

Additional file 2: **Table S2.** List of DEGs between Y35AB vs CK. (XLS 796 kb)

Additional file 3: **Table S3.** List of DEGs between Y35A vs CK. (XLS 629 kb)

Additional file 4: **Table S4.** Enriched GO terms of DEGs between Y35AB vs CK. (XLS 3.15 mb)

Additional file 5: **Table S5.** Enriched GO terms of DEGs between Y35A vs CK. (XLS 2.83 mb)

Additional file 6: **Table S6.** Enriched KEGG pathways of DEGs between Y35AB vs CK. (XLS 216 kb)

Additional file 7: **Table S7.** Enriched KEGG pathways of DEGs between Y35A vs CK. (XLS 181 kb)
