# Supplementary material for: Transcriptome analysis of Nicotiana benthamiana infected by Tobacco curly shoot virus
Source: Virol J. 2018 Sep 3;15:138. doi: 10.1186/s12985-018-1044-1 (PMC6122796; doi:10.1186/s12985-018-1044-1)
Supplement: Supplementary file 1 — Table S1. Primers used for RT-qPCR. (DOCX 18.9 kb) [file 12985_2018_1044_MOESM1_ESM.docx]

Table S1 Primers used for RT-qPCR

| **Primer name** | **Forward primer sequence (5'-3')** | **Reverse primer sequence (5'-3')** | **Product length (bp)** |
| --- | --- | --- | --- |
| PasFa-F/PasFa-R | GTGCTCCTGCCCTTGCTATTAAG | CTTCCAACCCATCCAATCCAACC | 199 |
| GRP3a-F/GRP3a-R | CTTCCACCACTTCCGCAGAGG | TCCGCCGCCAGGGTATCC | 146 |
| CAX3-F/CAX3-R | GCTCATTGCCTACCTCGCTTAC | CACTGCTTCCTCTTCTTCCACTG | 115 |
| PasFb-F/PasFb-R | AGTGCTCCTGCCCTTGCTATC | TCCAACCCACCCAATCCAACC | 198 |
| GDCSa-F/GDCSa-R | GGTTTGGAGTGCCTATGGGTTATG | TAGCCTTGTCTCTGCGGATGTG | 177 |
| GRP-F/GRP-R | ATACTGCCGCTATGGTTGTTGC | GGCTTCCCTTCAGTCACTTTATCC | 102 |
| GDCSb-F/GDCSb-R | TGAAAGTGAAAGCAAGGCGGAAC | TGAGCATTGATGGTGGATGAGGAG | 143 |
| glsF-F/glsF-R | CCAGAGGACGCCACCATAGTAG | TTTCCAAGCACCACAACACACC | 185 |
| PasFc-F/PasFc-R | AGTGCTCCTGCCCTTGCTATC | TCCAACCCACCCAATCCAACC | 198 |
| PU-F/PU-R | AGAAGCAAAAGCAAGCAGACAATG | GCTGGCTCCAACATTAGTTCCG | 185 |
| PsaNa-F/PsaNa-R | TGCTGCTTCCTCCAACTCCTC | TGTGCCAAACTGAACTGTATATGC | 109 |
| PsaNb-F/PsaNb-R | CGCTGCTTCCTCCAACTCCTC | TGTGCCAAACTGAACTGTGTATGC | 148 |
